# Precise mapping of two conserved minimal epitopes on the nucleocapsid protein of porcine epidemic diarrhea virus

**DOI:** 10.3389/fmicb.2026.1871079

**Published:** 2026-07-21

**Authors:** Youhao Bu, Ruiqin Zhu, Ao Jiang, Yanze Li, Liuyang Jiao, Xun Huang, Lu Xia, Hui Hu, Gaiping Zhang, Yanan Wu

**Affiliations:** 1 International Joint Research Center of National Animal Immunology, College of Veterinary Medicine, Henan Agricultural University, Zhengzhou, China; 2 Ministry of Education Key Laboratory for Animal Pathogens and Biosafety, College of Veterinary Medicine, Henan Agricultural University, Zhengzhou, China; 3 Longhu Laboratory, Zhengzhou, China; 4 School of Advanced Agricultural Sciences, Peking University, Beijing, China

**Keywords:** epitope mapping, monoclonal antibody, nucleocapsid protein, porcine epidemic diarrhea virus (PEDV), truncation strategy

## Abstract

**Introduction:**

Porcine epidemic diarrhea virus (PEDV) is a highly contagious enteric pathogen that causes severe diarrhea and high mortality in neonatal piglets, leading to substantial economic losses in the swine industry. The nucleocapsid (N) protein is highly conserved and abundantly expressed during infection, making it a suitable target for serological detection.

**Methods:**

In this study, twomonoclonal antibodies (mAbs), C9 and G11, directed against the PEDV N protein were used to define minimal linear epitopes. A stepwise truncation approach based on overlapping recombinant fragments was applied to localize the antigenic regions.

**Results:**

The results showed that mAb C9 and mAb G11 recognized the minimal epitopes ^58^RWRM^61^ and ^15^PLSL^18^, respectively. Sequence analysis indicated that both epitopes are highly conserved among representative PEDV strains from different genotypes. In addition, structural modeling suggested that these regions are exposed on the surface of the N protein, consistent with their accessibility to antibody binding.

**Discussion:**

These findings provide a more precise characterization of antigenic sites within the PEDVNprotein andmay be useful for the development of specific and reliable serological assays.

## 1 Introduction

Porcine epidemic diarrhea virus (PEDV), a member of the genus Alphacoronavirus within the family Coronaviridae (order Nidovirales), is the causative agent of porcine epidemic diarrhea (PED), a highly contagious enteric disease characterized by severe diarrhea, vomiting, dehydration, and high mortality in neonatal piglets (Jung et al., 2020; Pensaert and de Bouck, 1978; Zhang et al., 2022; Schulz and Tonsor, 2015). Since its first identification in Europe in the 1970s, PEDV has spread widely across major swine-producing regions (Zhang et al., 2023; Li et al., 2018). Although the disease was brought under control in some areas, the emergence of highly virulent variants after 2010 has led to large-scale outbreaks in Asia and North America, resulting in considerable economic losses to the swine industry (Li et al., 2025a; Turlewicz-Podbielska and Pomorska-Mól, 2021; Lin et al., 2022).

PEDV is an enveloped, single-stranded, positive-sense RNA virus with a genome of approximately 28 kb, encoding four structural proteins—spike (S), envelope (E), membrane (M), and nucleocapsid (N)—as well as multiple non-structural and accessory proteins (Hanna and Małgorzata, 2021). Among these, the spike protein has been extensively studied because of its role in receptor binding and induction of neutralizing antibodies (Polyiam et al., 2021; Wang et al., 2026). In contrast, the nucleocapsid (N) protein has received comparatively less attention, despite being one of the most abundant viral proteins and highly conserved among PEDV strains. The N protein is expressed early during infection and participates in several steps of the viral life cycle, including RNA binding, genome encapsidation, and regulation of viral replication and transcription (Zhang et al., 2022; Li et al., 2020). It has also been implicated in modulating host innate immune responses, suggesting that its antigenic regions may reflect both structural and functional constraints (Han et al., 2024). Owing to its high abundance and strong immunogenicity, the N protein has been widely used as a target antigen in serological assays (Zhai et al., 2023). Compared with the S protein, which is prone to genetic variation, the relatively conserved nature of the N protein makes it more suitable for detecting diverse PEDV strains (Lei et al., 2024). However, a critical research gap remains in the current understanding of the antigenicity of the PEDV N protein: although several antigenic regions of the N protein have been identified, nearly all previous epitope mapping studies have relied on longer peptide fragments. The frequent non-specific cross-reactions inherent in these long antigenic sequences can compromise the diagnostic specificity of N protein-based serological assays (Hao et al., 2022).

To overcome this limitation, there is an urgent need to precisely identify the smallest linear epitopes. This not only deepens our understanding of the mechanisms underlying antibody-antigen interactions at the amino acid resolution level but also yields tangible practical benefits (Fung and Liu, 2018; Huang et al., 2004; McBride et al., 2014; Takeda et al., 2008). Short minimal peptide epitopes offer significant technical and economic advantages: they can be easily synthesized chemically and readily purified to high purity. More importantly, short conserved epitopes effectively reduce off-target binding signals and substantially improve diagnostic specificity (Hao et al., 2025). Conservative minimal epitopes can also serve as powerful universal biomarkers for tracking genetically heterogeneous viral variants, a feature of great significance for large-scale global PEDV surveillance programs (Zhang et al., 2022; Xie et al., 2019).

In this study, the present study aimed to systematically characterize the minimal linear epitopes recognized by two monoclonal antibodies specific to the PEDV N protein. Through iterative truncation screening of overlapping recombinant N protein fragments, we successfully identified two ultra-short core epitope motifs consisting of only four amino acids (^58^RWRM^61^ and ^15^PLSL^18^), which provide a viable strategy for improving the specificity of PEDV serological testing. We further evaluated the cross-strain conservation of these two epitopes and visualized their spatial localization within the N protein through structural modeling. Overall, our findings refine the antigenic map of the PEDV nucleocapsid protein and lay a foundation for the development of standardized, highly specific serological detection tools suitable for global, unified PEDV surveillance.

## 2 Materials and methods

### 2.1 Materials and reagents

The pGEX-6p-1 vector, the full-length PEDV N protein expression plasmid (pET28a-N), and the hybridoma cell lines producing monoclonal antibodies C9 and G11 were maintained in the laboratory. Hybridoma serum-free medium was purchased from Yuanpei Biotech (Shanghai, China). *Escherichia coli* DH5α and B21(DE3) strains were purchased from Tsingke Biotechnology (Beijing, China). Rapid protein transfer buffer was obtained from Servicebio (Wuhan, China). ECL detection reagents and HRP-conjugated goat anti-mouse IgG (H + L) were obtained from Xinsaimai. Restriction enzymes *Bam*HI and *Eco*RI were sourced from New England BioLabs (Ipswich, MA, USA). The homologous recombination kit was obtained from Novazene (Nanjing, China), and isopropyl β-D-thiogalactoside (IPTG) was purchased from Solarbio Science & Technology (Beijing, China).

### 2.2 Expression and purification of recombinant N protein

Based on the PEDV N gene sequence (GenBank accession number: MT338518.1), the upstream primer 5′-CGCGGCAGCCATATGATGGCTTCTGTCAGTTTTCA-3′ (F) and the downstream primer 3′-GGTGGTGGTGCTCGATTAATTTCCTGTATCGAAGATCT-5′ were designed. Using the PEDV HNAY strain as a template, reverse transcription of RNA was performed, followed by amplification of the PEDV N gene. The amplified N gene was inserted into the pET28a vector using the NdeI and XhoI restriction enzyme sites to construct the recombinant plasmid. The correct plasmid was transformed into BL21 (DE3) cells, which were then treated with 0.1 mM isopropyl-β-D-1-thio-galactoside (IPTG) for 4 h at 37 °C. Protein expression was analyzed by SDS-PAGE; the protein was purified using nickel-nitrotriacetic acid (Ni-NTA) metal affinity chromatography; and the reactivity of the purified protein was verified by Western blot.

### 2.3 Preparation of monoclonal antibody supernatant

Take 30 μg of purified N-protein antigen per mouse and thoroughly vortex-mix it with QuickAntibody-Mouse5W adjuvant in a 1:1 volume ratio. Administer the mixture via multipoint subcutaneous injection into the backs of female BALB/c mice on days 0 and 21 of the immunization schedule. On day 35, blood was collected from the tail veins of the mice, and serum antibody titers were determined using an indirect ELISA to screen for mice with high titers. Positive mice received a booster immunization via intraperitoneal injection of 20 μg of unadjuvanted purified N-protein. 3 days after the booster, splenic cells were aseptically isolated from the mice and fused with SP2/0 myeloma cells via PEG-mediated cell fusion. After plating the fusion cells, they were cultured in HAT selective medium, and hybridoma-positive wells secreting N-protein-specific antibodies were screened using indirect ELISA; three rounds of subcloning were conducted consecutively using the limited dilution method, with ELISA re-testing after each round to eliminate negative cell wells, ultimately yielding a stable hybridoma cell line secreting specific monoclonal antibodies. The antibody subtype of the positive hybridoma was determined using a mouse monoclonal antibody subtype identification kit.

Hybridoma cells were rapidly thawed in a 37 °C water bath and immediately transferred to centrifuge tubes. After centrifugation at 600 × g for 5 min, the supernatant was discarded, and the cell pellet was resuspended in serum-free hybridoma medium. Cells were then cultured in T75 flasks at 37 °C in a humidified incubator with 5% CO_2_. Culture supernatants were collected after 2–3 days and stored at −20 °C.

### 2.4 Indirect ELISA

First, the optimal protein coating concentration was determined using a grid method. The protein was diluted from 100 ng/well to 1000 ng/well and used to coat the microplate. The plate was incubated at 37 °C for 2 h and washed three times with PBST. Block with 5% skim milk and incubate at 37 °C for 1 h, then wash three times with PBST. Dilute mouse serum at a ratio of 1:100 to 1:1000, add 100 μL to each well, incubate at 37 °C for 1 h, and wash five times with PBST. Add horseradish peroxidase (HRP)-labeled secondary antibody (goat anti-mouse IgG), incubate at 37 °C for 45 min, and wash five times with PBST. Add the color development solution, place the plate in the dark, and let it stand for 15 min. Add the stop solution, and measure the OD450 value using an enzyme microplate reader.

### 2.5 Western blot analysis

Recombinant proteins were separated by 10% sodium dodecyl sulfate polyacrylamide gel electrophoresis (SDS-PAGE). Under conditions of 400 mA for 30 min, the solution was transferred onto a polyvinylidene fluoride (PVDF) membrane (C3117, Millipore, Massachusetts, USA) with a pore size of 0.45 μm. The PVDF membrane was blocked with 5% skim milk in TBST at room temperature for 2 h and incubated overnight at 4 °C with monoclonal antibody supernatants (C9 or G11; no dilution required). For control, GST protein purified from the same batch was used to ensure that the amount of GST protein loaded at different truncation time points was consistent, membranes were incubated with anti-GST antibody (1:5000). After three washes with TBST, membranes were incubated with HRP-conjugated goat anti-mouse IgG (1:5000) for 45 min at room temperature. Signals were visualized using an enhanced chemiluminescent system.

### 2.6 IFA antibody characterization report

Culture Vero cells in a 24-well plate until a monolayer forms. Wash 2 to 3 times with PBS, then inoculate with PEDV. As soon as any cytopathic effects are observed, immediately discard the culture medium and wash 2 to 3 times with PBS. Subsequently, fix the cells with 4% paraformaldehyde at room temperature for 30 min, then permeabilize them with 0.1% Triton X-100 at room temperature for 15 min; block with 5% bovine serum albumin (BSA) at room temperature for 1 h; incubate with hybridoma supernatant as the primary antibody at room temperature for 1 h; FITC-labeled goat anti-mouse IgG (1:500) was diluted and incubated for 1 h at room temperature in the dark. Subsequently, the cells were stained with DAPI for 10 min in the dark, and washed three times with PBS and ddH_2_O, respectively. Observations were made using an LSM 800 laser-scanning confocal microscope.

### 2.7 Identification of B-cell epitopes

Preliminary experiments indicated that monoclonal antibodies C9 and G11 recognize linear epitopes on the N protein. In this experiment, the N protein was truncated in five rounds to further determine the minimal linear epitopes recognized by these two monoclonal antibodies. Using the full-length pET28a-N plasmid as a template and BamHI and EcoRI as restriction sites, a total of 33 gene fragments were amplified. Primer information is shown in Tables 1, 2. The amplification products were inserted into the pGEX-6p-1 vector via homologous recombination, and the ligation products were transformed into DH5α competent cells. Small-scale plasmid transformation was performed on strains with verified sequences, and the recombinant plasmids were transformed into *E. coli* BL21 (DE3). Expression was carried out for 5 h at 37 °C in the presence of 1 mM isopropyl-β-D-thio-galactoside (IPTG), resulting in the expression of the target antigen as a GST fusion protein. After purification of the recombinant protein, it was identified using Western blot analysis.

**Table 1:** Primers used for amplification of truncated fragments for epitope mapping of mAb C9.

Primer	Sequence (5′-3′)
AA1-157-F	GCCCCTGGGATCCATGGCCCCTGTCAGTT
AA1-157-R	GTCGACCCGGGAATTCGTTGTTGCCATTACC
AA143-299-F	GGCCCCTGGGATCCTCACGTGCAAATTCAC
AA143-299-R	TCGAGTCGACTCCCCTGGGTCCGAAG
AA285-441-F	TTCCAGGGGCCCCTGGGATCCCCCAAGGGCGAAAATA
AA285-441-R	GCTCGAGTCGACCCGGGAATTCTTAATTTCCTGTATCG
AA1-62-F	GGGCCCCTGGGATCCATGGCTTCTGTCAGTTTT
AA1-62-R	CCGGGAATTCGCGCATGCGCCAGCGAATTT
AA47-62-F	GCCCCTGGGATCCGACCAGCAAATTGGGTACT
AA47-62-R	CGAGTCGACCCGGGAATTCATTAGTGGGTTCAGTC
AA94-157-F	CCCCTGGGATCCGGTGTTTTCTGGGTT
AA94-157-R	CGACCCGGGAATTCGTTGTTGCCATTACC
AA48-62-F	GGGCCCCTGGGATCCCAGCAAATTGGGTAC
AA48-62-R	CGAGTCGACCCGGGAATTCATTAGTGGGTTCAGTC
AA49-62-F	GGGCCCCTGGGATCCCAAATTGGGTACTGG
AA49-62-R	CGAGTCGACCCGGGAATTCATTAGTGGGTTCAGTC
AA50-62-F	GGGCCCCTGGGATCCATTGGGTACTGGAAT
AA50-62-R	CGAGTCGACCCGGGAATTCATTAGTGGGTTCAGTC
AA51-62-F	GGGCCCCTGGGATCCGGGTACTGGAATGAG
AA51-62-R	CGAGTCGACCCGGGAATTCATTAGTGGGTTCAGTC
AA52-62-F	AGGGGCCCCTGGGATCCTACTGGAATGAGC
AA52-62-R	CGAGTCGACCCGGGAATTCATTAGTGGGTTCAGTC
AA53-62-F	GGGGCCCCTGGGATCCTGGAATGAGCAAAT
AA53-62-R	CGAGTCGACCCGGGAATTCATTAGTGGGTTCAGTC
AA54-62-F	GGGGCCCCTGGGATCCAATGAGCAAATTCGC
AA54-62-R	CGAGTCGACCCGGGAATTCATTAGTGGGTTCAGTC
AA55-62-F	GGGCCCCTGGGATCCGAGCAAATTCGCTG
AA55-62-R	CGAGTCGACCCGGGAATTCATTAGTGGGTTCAGTC
AA56-62-F	GGGGCCCCTGGGATCCCAAATTCGCTGGCG
AA56-62-R	CGAGTCGACCCGGGAATTCATTAGTGGGTTCAGTC
AA57-62-F	GGCCCCTGGGATCCATTCGCTGGCGCAT
AA57-62-R	CGAGTCGACCCGGGAATTCATTAGTGGGTTCAGTC
AA58-62-F	GGCCCCTGGGATCCCGCTGGCGCAT
AA58-62-R	CGAGTCGACCCGGGAATTCATTAGTGGGTTCAGTC
AA1-59-F	GGGCCCCTGGGATCCATGGCTTCTGTCAGTTTT
AA1-59-R	CGAGTCGACCCGGGAATTCCCAGCGAATTTGCTC
AA1-60-F	GGGCCCCTGGGATCCATGGCTTCTGTCAGTTTT
AA1-60-R	TCGACCCGGGAATTCGCGCCAGCGAATTTG
AA1-61-F	GGGCCCCTGGGATCCATGGCTTCTGTCAGT
AA1-61-R	TCGACCCGGGAATTCCATGCGCCAGCGAAT

**Table 2:** Primers used for amplification of truncated fragments for epitope mapping of mAb G11.

Primer	Sequence (5^′^-3^′^)
AA1-31-F	GCCCCTGGGATCCATGGCTTCTGTCAGTTT
AA1-31-R	TCGACCCGGGAATTCAGAAAGGGGTTTGTC
AA15-46-F	GGGCCCCTGGGATCCCCATTATCCCTCTAT
AA15-46-R	CGAGTCGACCCGGGAATTCCTTATTTCCTT
AA30-62-F	GGGCCCCTGGGATCCCTTTCTAAGGTACTT
AA30-62-R	ACCCGGGAATTCCATGCGCCAGCGAATTTG
AA1-10-F	GGGCCCCTGGGATCCATGGCTTCTGTCAGT
AA1-10-R	GAGTCGACCCGGGAATTCGCCACGATCCTG
AA3-12-F	GGGCCCCTGGGATCCTCTGTCAGTTTTCAG
AA3-12-R	TCGACCCGGGAATTCTTTGCGGCCACGATC
AA5-14-F	GGGCCCCTGGGATCCAGTTTTCAGGATCGT
AA5-14-R	GAGTCGACCCGGGAATTCCACCCGTTTGCG
AA7-16-F	GGGCCCCTGGGATCCCAGGATCGTGGC
AA7-16-R	CGAGTCGACCCGGGAATTCTAATGGCACCCG
AA9-18-F	CCCCTGGGATCCCGTGGCCGCAAAC
AA9-18-R	GTCGACCCGGGAATTCGAGGGATAATGGC
AA11-20-F	CCCCTGGGATCCCGCAAACGGGTGCC
AA11-20-R	AGTCGACCCGGGAATTCGGCATAGAGGGAT
AA13-22-F	GGCCCCTGGGATCCCGGGTGCCATTAT
AA13-22-R	AGTCGACCCGGGAATTCAAGAGGGGCATAG
AA14-31-F	CCCCTGGGATCCGTGCCATTATCCCTCTAT
AA14-31-R	TCGACCCGGGAATTCAGAAAGGGGTTTGTC
AA1-17-F	GGGCCCCTGGGATCCATGGCTTCTGTCAGT
AA1-17-R	TCGACCCGGGAATTCGGATAATGGCACCCG

The truncated sequences used in the first two rounds of epitope mapping were identical; the corresponding primer sequences are listed in Table 1.

### 2.8 Epitope conservation analysis

To evaluate the conservation of epitope, Clustal Omega (https://www.ebi.ac.uk/Tools/msa/clustalo/) was used to align the sequences containing ^58^RWRM^61^ and ^15^PLSL^18^ with 25 porcine epidemic diarrhea virus (PEDV) strains randomly selected from relevant literature. Sequences were retrieved from GenBank (https://www.ncbi.nlm.nih.gov/gene/), with details provided in Table 3.

**Table 3:** PEDV strains used for sequence conservation analysis in this study.

Strains	Country	Accession number	Subtype or Characteristic
HNAY2016	China	MT338518.1	GIIa
CV777	Belgium	AF353511.1	GIa
CHM2013	China	KM887144.1	GIa
CH/S	China	JN547228.1	GIa
DR13	South Korea	JQ023161.1	GIa
LZC	China	EF185992.1	GIa
SD-M	China	JX560761.1	GIb
JS-2004-2	China	AY653206.1	GIb
SC1402	China	KP162057.1	GIb
SQ2014	China	KP728470.1	GIb
AH-M	China	KJ158152.1	GIb
GD-B	China	JX088695.1	GIIa
AH2012/12	China	KU646831.1	GIIa
LZW	China	KJ777678.1	GIIa
CH/ZMDZY/11	China	KC196276	GIIa
CH/FJZZ-9/2012	China	KC140102.1	GIIa
AJ1102	China	JX188454.1	GIIb
CHGD-01	China	JX261936.1	GIIb
GD-A	China	JX112709.1	GIIb
LC	China	JX489155.1	GIIb
ZJCZ4	China	JX524137.1	GIIb
OH851	USA	KJ399978.1	GIIc
CH/HNQX-3/14	China	KR095279.1	GIIc
KNU-1406-1	South Korea	KM403155.1	GIIc
TC Iowa106(PV39)-P1	USA	KM392232.1	GIIc
15V010/BEL/2015	Belgium	KR003452.1	GIIc

### 2.9 Structural analysis of epitopes

The three-dimensional structure of the PEDV N protein was predicted using the I-TASSER server (Zheng et al., 2021; Roy et al., 2010). The spatial distribution of the epitopes was visualized using PyMOL 3.0.

## 3 Results

### 3.1. Expression and purification of recombinant N protein

**Figure 1:**
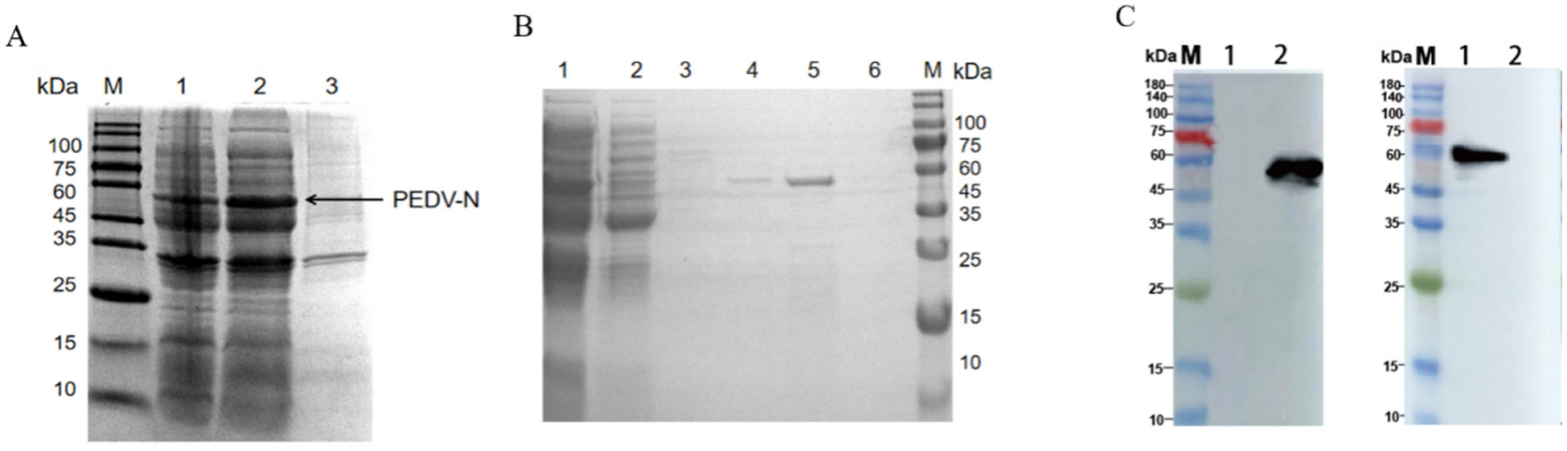
Expression and purification of PEDV N protein and analysis of protein immunogenicity. **(A)** SDS-PAGE was used to analyze the expression of the recombinant N protein. Lane 1: Uninduced bacterial culture; Lane 2: Supernatant after lysis; Lane 3: Precipitate after lysis. **(B)** SDS-PAGE was used to analyze the purification of N protein using a nickel column. Lane 1: Permeating; Lanes 2–6: 20 mm imidazole, 50 mm imidazole, 100 mm imidazole, 300 mm imidazole, 500 mm imidazole elution solutions. **(C)** Western blot analysis of whether N protein was correctly expressed **(left)** and immunogenicity **(right)**. The pET28a empty vector was used as a negative control.

The size of the recombinant N protein is approximately 58 kDa, and it is expressed as a soluble protein in the supernatant (Figure 1A). It was purified on a nickel-triacetic acid affinity chromatography column and batch eluted at a 300 mM imidazole concentration (Figure 1B). Subsequently, Western blot verification was performed on the purified protein using anti-His tag antibody and PEDV positive serum (Figure 1C). The anti-His antibody indicated that the recombinant protein was correctly expressed and purified, and the PEDV positive serum confirmed the good immunogenicity of the recombinant protein.

### 3.2 . Determination of the optimal antigen coating concentration

For the subsequent screening of monoclonal antibodies, we determined the optimal protein coating amount for indirect ELISA using the checkerboard method. The results of the checkerboard experiment showed that when the serum dilution was 1:800 and the protein coating amount was 600 ng/well, the P/N value was the highest (Table 4). Therefore, the ELISA experiment was conducted with a coating amount of 600 ng/well in the subsequent experiments.

**Table 4:** Determination of the optimal dilution degrees of antigens and serum (P/N value).

Serum dilution ratio	PEDV N protein (ng/each well)					
	100	200	400	600	800	1000
1:200	6.35	5.75	5.46	8.04	8.20	6.67
1:400	10.65	9.47	8.19	13.90	14.15	11.15
1:600	13.83	14.62	12.44	19.01	17.58	17.01
1:800	18.85	18.73	14.36	26.04	24.25	19.06
1:1000	15.09	15.52	11.98	22.65	21.51	19.351

### 3.3 . Characterization of N protein-specific monoclonal antibodies

**Figure 2:**
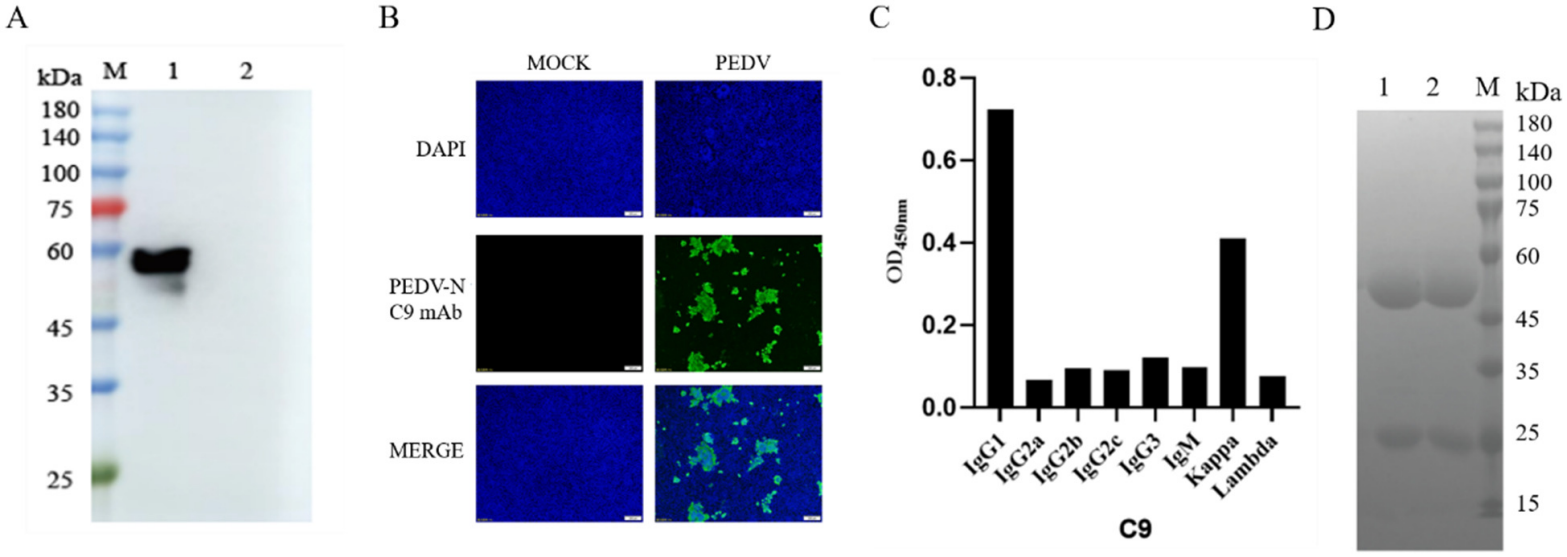
Characterization and purification of mAb. **(A)** Western blot was used to analyze the reactivity of mAb C9 with the recombinant protein. Lane 1: N protein; Lane 2: negative control. **(B)** IFA was used to analyze the reactivity of mAb C9 to the PEDV strain. **(C)** Subtype identification of mAb C9. **(D)** Purification of mAb C9 ascites.

After three rounds of subcloning, specific monoclonal antibodies against the N protein were screened out and named C9 and G11. The monoclonal antibodies were verified to have specific reactions with the recombinant protein through Western blot (Figure 2A). To further confirm the reactivity of the monoclonal antibodies to the PEDV virus, IFA tests were conducted, and the results showed that the monoclonal antibodies had good specificity signals for the virus (Figure 2B). The monoclonal antibodies were identified using the mouse monoclonal antibody subtype identification kit, and the heavy chains were all IgG1, and the light chains were all Kappa (Figure 2C). The monoclonal antibody ascites was purified using Protein G, and the SDS-PAGE results indicated that the purity of the monoclonal antibody ascites was very high (Figure 2D). The characterization of monoclonal antibody G11 has been presented in another article (Sun et al., 2025), and it will not be shown again here.

### 3.4 Generation of truncated constructs

**Figure 3:**
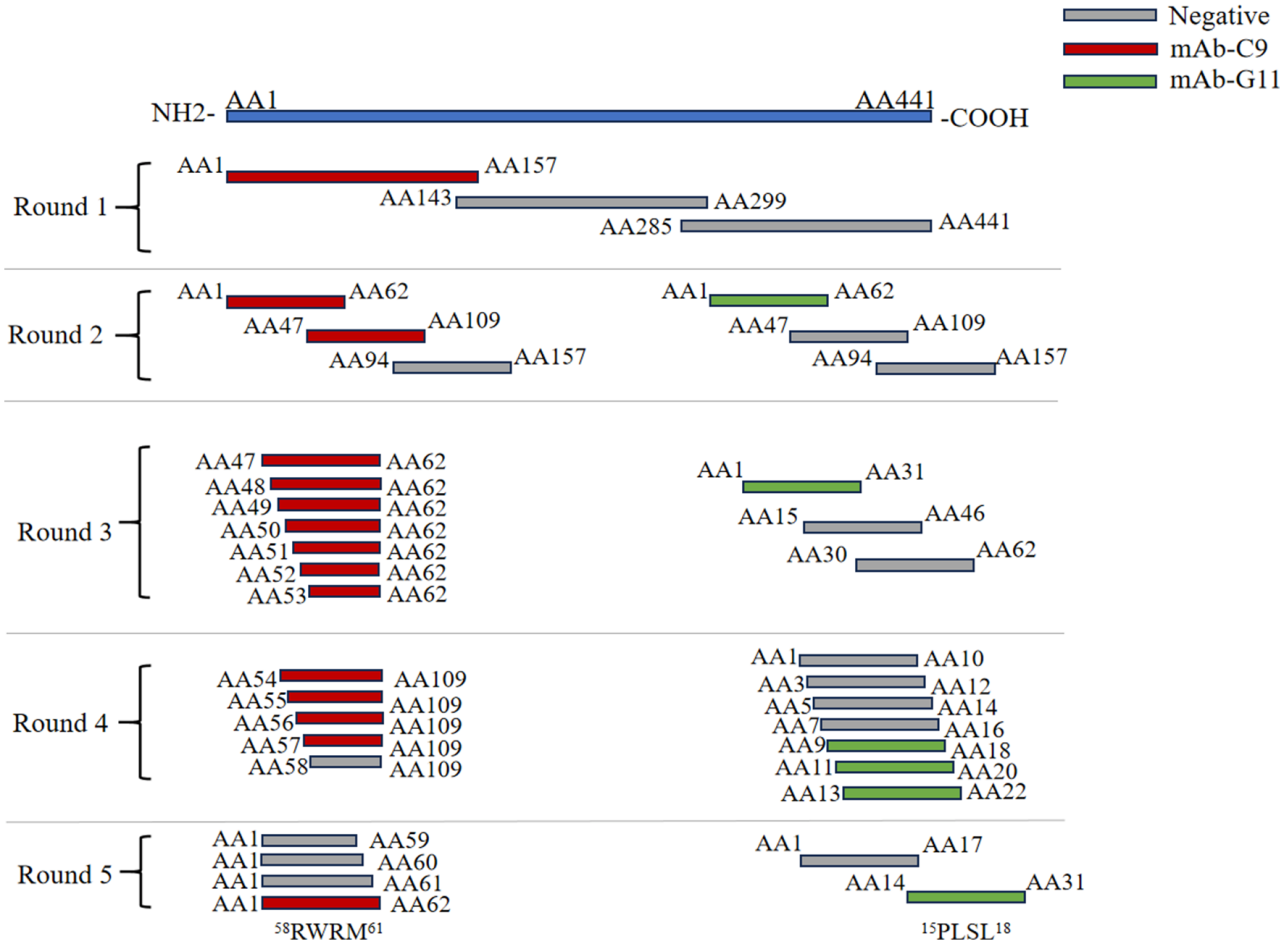
Schematic representation of the PEDV N protein and its truncated fragments used for epitope mapping. The full-length N protein (441 amino acids, AA) is shown in blue. Truncated fragments recognized exclusively by mAb C9 are highlighted in red, whereas those recognized exclusively by mAb G11 are shown in green. Fragments not recognized by either mAb C9 or mAb G11 are indicated in gray. The numbers represent the amino acids positions within the full-length N protein and each truncated fragment.

In this study, a truncation strategy was designed to identify minimal epitope regions: the N protein underwent five rounds of truncation to further determine the minimal linear epitope recognized by these two antibodies (Figure 3). The full-length PEDV N gene in pET28a-N was used as a template for PCR amplification. A total of 33 truncated fragments were generated using primers containing BamHI and EcoRI restriction sites (Tables 1, 2).

### 3.5 Identification of minimal epitopes recognized by mAbs C9 and G11

**Figure 4:**
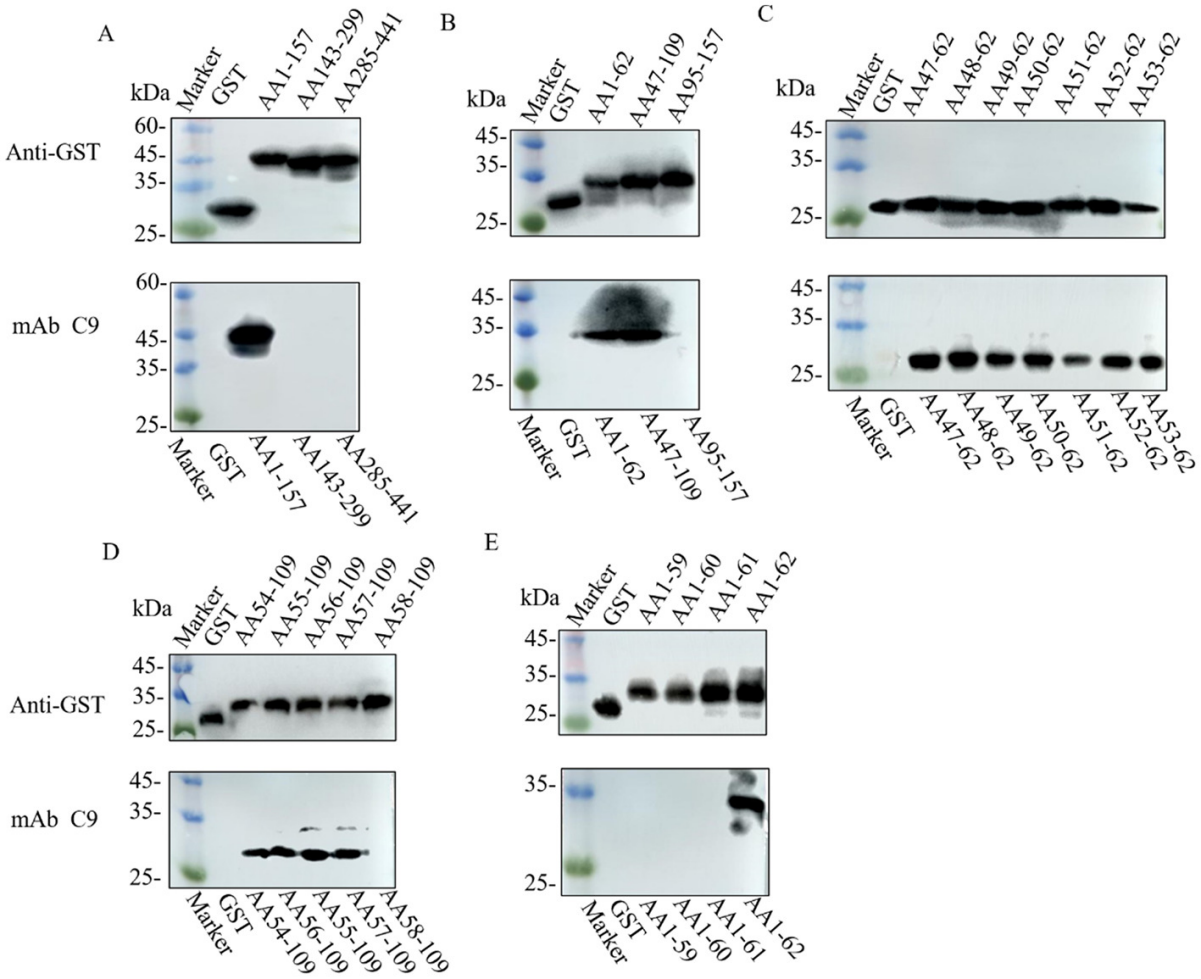
Mapping of the epitope recognized by mAb C9. **(A–E)** Sequential truncation analysis of the PEDV N protein. Reactivity of each fragment was evaluated by Western blot using mAb C9. Anti-GST antibody was used as a positive control. AA: amino acid.

**Figure 5:**
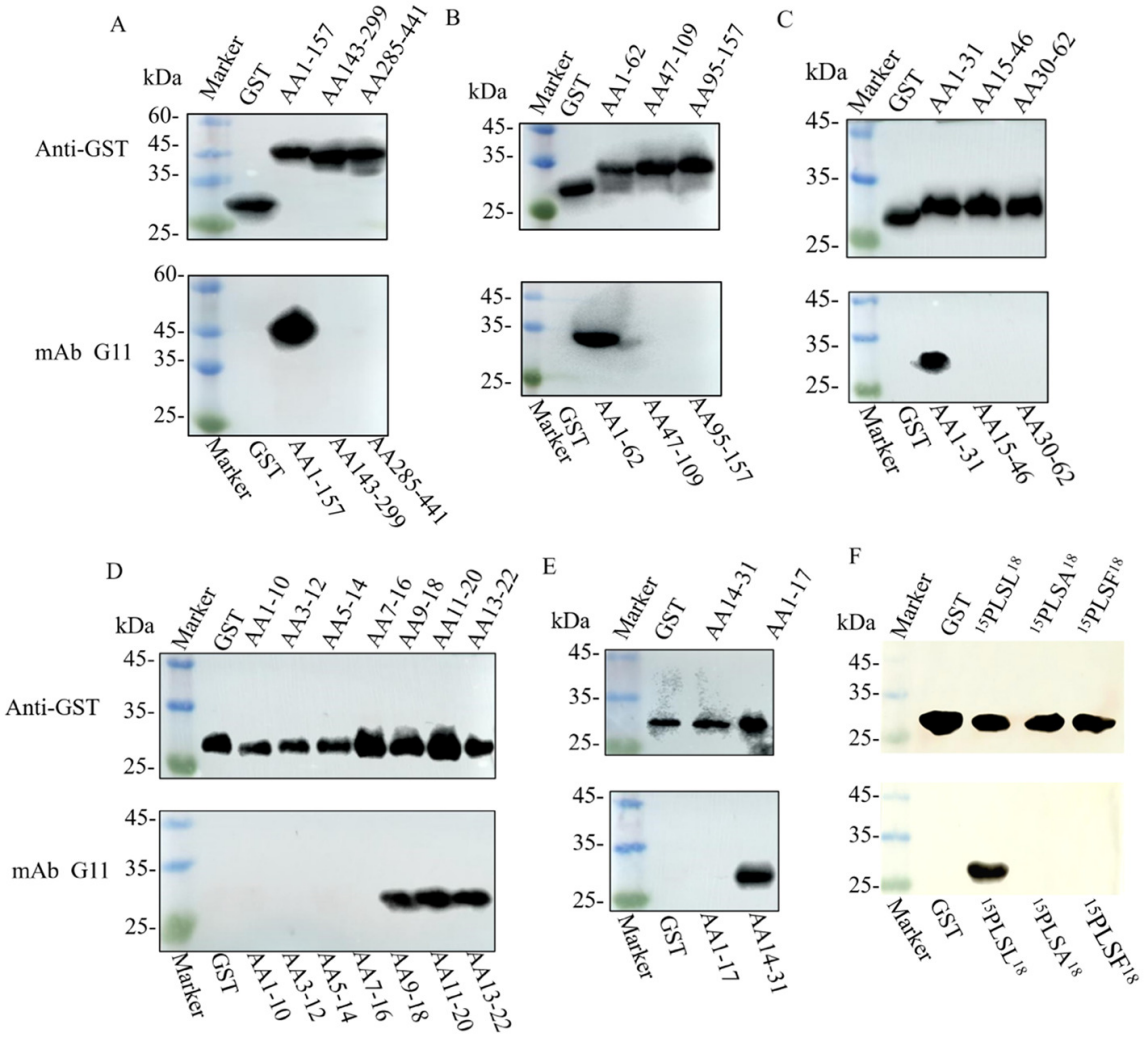
Mapping of the epitope recognized by mAb G11. **(A–E)** Sequential truncation analysis of the PEDV N protein; **(F)** Site-specific analysis of the L18 mutation to L18A and L18F. Reactivity of each fragment was evaluated by Western blot using mAb G11. Anti-GST antibody was used as a positive control. AA, amino acid.

To map the antigenic epitopes recognized by mAbs C9 and G11, the full-length PEDV N protein was initially divided into three overlapping fragments (aa1–157, aa143–299, and aa285–441). Western blot analysis showed that both mAbs specifically reacted with the aa1–157 fragment, while no reactivity was observed with the other two fragments (Figures 4A, 5A), indicating that the epitopes recognized by both antibodies are located within the N-terminal region of the N protein. To further localize the epitope recognized by mAb C9, the aa1–157 region was subdivided into smaller overlapping fragments. As shown in Figure 4B, mAb C9 reacted with fragments aa1–62 and aa47–109, suggesting that the epitope is located within the overlapping region aa47–62. To precisely define the minimal epitope, a series of sequential truncations was performed. N-terminal truncation analysis demonstrated that fragment aa53–62 retained full reactivity with mAb C9 (Figure 4C), indicating that residues upstream of position 53 are not essential for antibody recognition. However, when the truncation was extended to aa58–109, the reactivity was completely lost (Figure 4D), suggesting that key residues required for binding are located between aa53 and aa58. To further refine the epitope, C-terminal truncation was performed. The results showed that mAb C9 recognized fragment aa58–62 but failed to recognize shorter fragments lacking C-terminal residues (Figure 4E). Taken together, these results indicate that the minimal linear epitope recognized by mAb C9 is located at aa58–61 (^58^RWRM^61^).

For mAb G11, initial truncation analysis revealed that the antibody specifically recognized fragment aa1–31 (Figure 5B), indicating that its epitope is located within the extreme N-terminal region. Further truncation showed that mAb G11 did not react with fragment aa15–46 (Figure 5C), suggesting that the epitope is not located within aa15–31. To further narrow down the epitope region, a panel of overlapping short peptides (aa1–10, aa3–12, aa5–14, aa7–16, aa9–18, aa11–20, and aa13–22) was constructed and analyzed. As shown in Figure 5D, mAb G11 reacted with fragments aa9–18, aa11–20, and aa13–22, but not with the other fragments, indicating that the core epitope is located within aa13–18. To precisely define the minimal epitope, two additional truncation constructs (aa14–31 and aa1–17) were generated. The results showed that mAb G11 recognized aa14–31 but failed to recognize aa1–17 (Figure 5E), indicating that residues within aa15–18 are critical for antibody binding. Therefore, the minimal linear epitope recognized by mAb G11 was determined to be aa15–18 (^15^PLSL^18^). To verify whether the L18F substitution (PLSF motif) in certain variants would weaken or completely eliminate the recognition ability of mAb G11, we introduced L18A and L18F point mutations based on AA9–18 (Figure 5F).The results indicate that the 18th amino acid is indeed essential for binding; however, the L18F mutation virtually eliminated mAb G11′s recognition ability, which imposes certain limitations on the assay.

Together, these data define the minimal linear epitopes recognized by mAbs C9 and G11 and provide a clear reference for subsequent analyses.

### 3.6 Conservation analysis of identified epitopes

**Figure 6:**
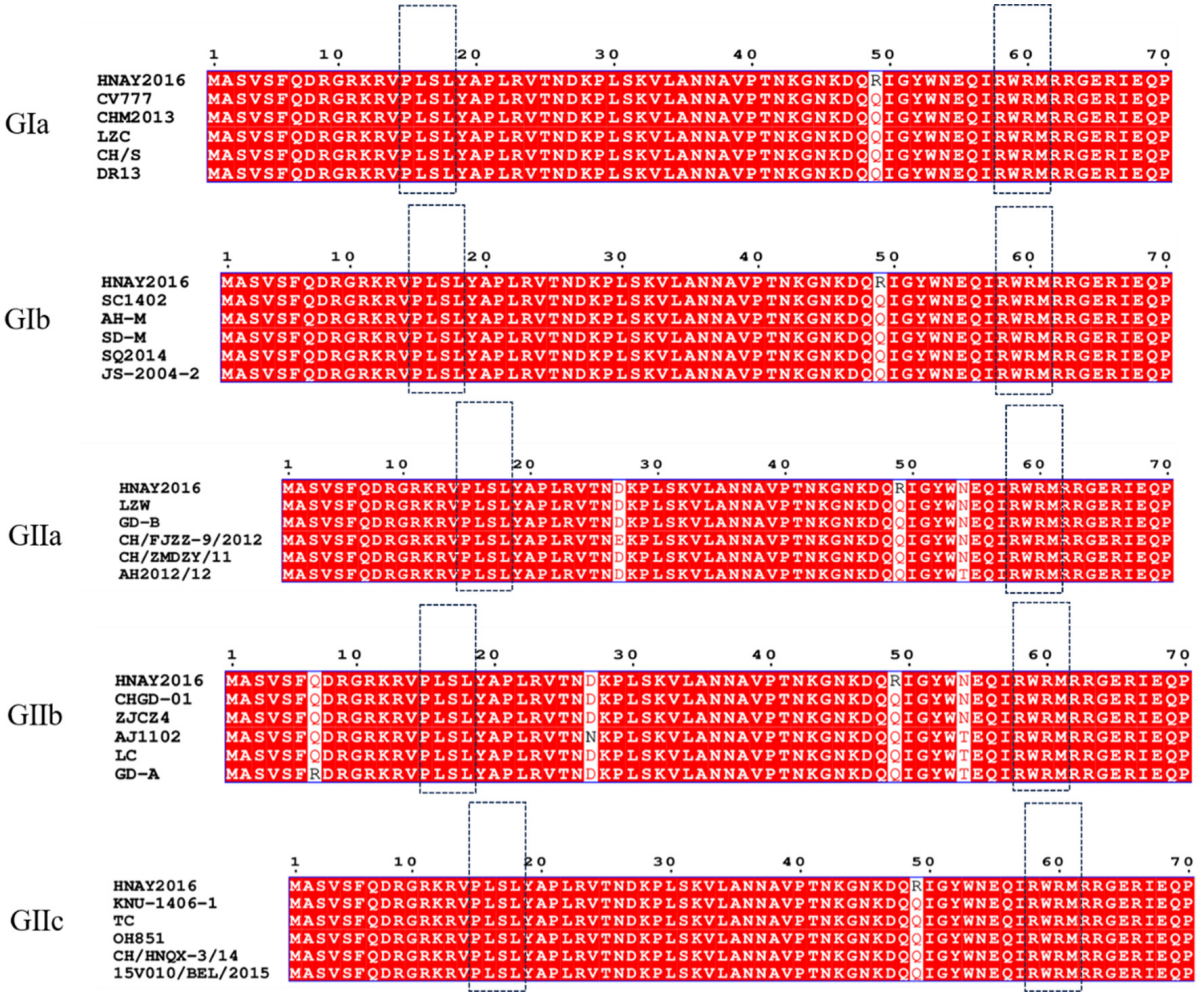
Conservation analysis of identified B-cell epitopes. Multiple sequence alignment of PEDV N protein regions containing the epitopes ^58^RWRM^61^ and ^15^PLSL^18^ across 25 representative strains. The HNAY2016 strain was used as the reference sequence, and only residues corresponding to the epitope regions are shown.

To evaluate the conservation of the identified epitopes among different PEDV strains, sequence alignment was performed using 25 reference strains representing multiple PEDV genotypes. The amino acid sequences corresponding to the two epitopes (^58^RWRM^61^ and ^15^PLSL^18^) were extracted and compared. As shown in Figure 6, although the selected strains belong to five distinct subtypes, both epitopes exhibited a high degree of conservation, with no amino acid substitutions observed in the aligned regions. These results indicate that the identified epitopes are highly conserved among diverse PEDV strains. The conservation of these epitopes across different PEDV genotypes indicates that they remain relatively stable and may be suitable targets for broad detection of PEDV strains.

### 3.7 Structural localization of identified epitopes

**Figure 7:**
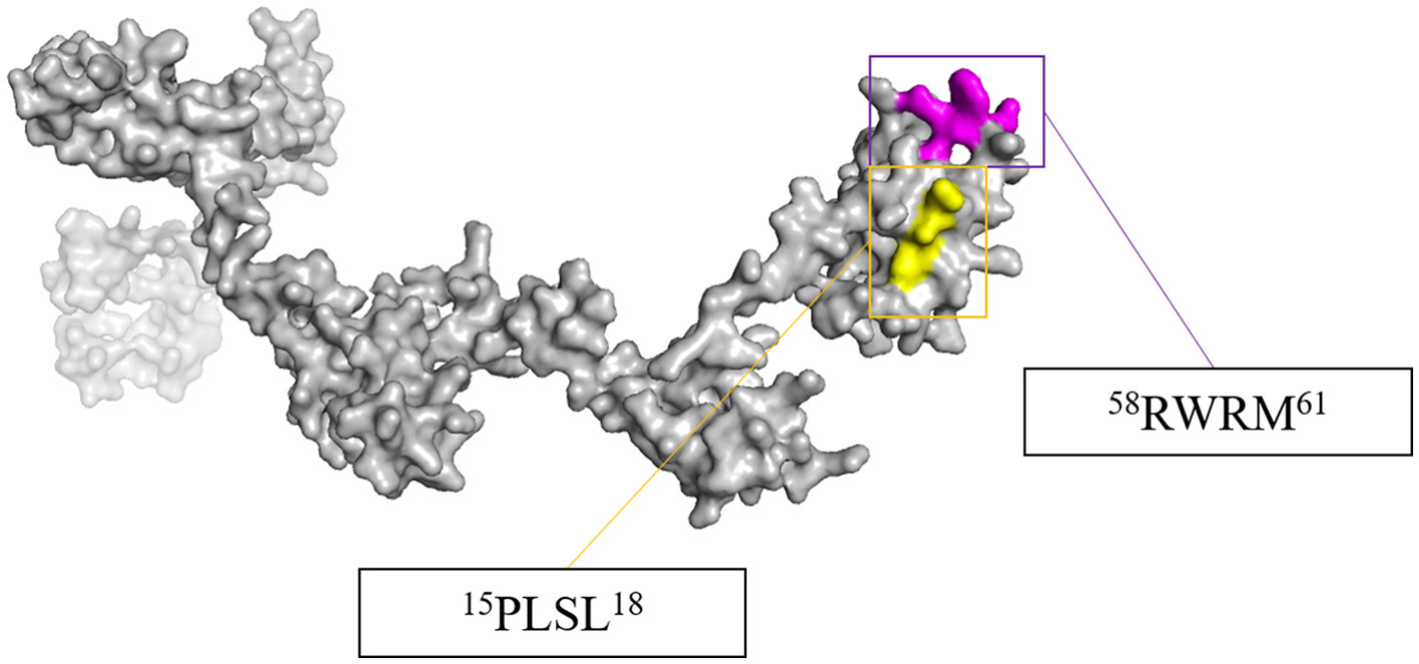
Structural localization of identified B-cell epitopes. The predicted three-dimensional structure of the PEDV N protein showing the spatial distribution of epitopes ^58^RWRM^61^ (purple) and ^15^PLSL^18^ (yellow) on the protein surface.

To investigate the spatial distribution of the identified epitopes, a three-dimensional structural model of the PEDV N protein was generated using the I-TASSER server. The positions of the epitopes were subsequently visualized using PyMOL software. As shown in Figure 7, both epitopes (^58^RWRM^61^ and ^15^PLSL^18^) are located on the surface of the N protein. This surface exposure suggests that these regions are accessible for antibody binding, which is consistent with their identification as B-cell epitopes. Their surface exposure is consistent with antibody accessibility and supports their relevance as B-cell epitopes in diagnostic settings.

## 4 Discussion

Porcine epidemic diarrhea virus (PEDV) remains a major enteric pathogen in swine, particularly affecting neonatal piglets with high mortality (Wang et al., 2025b; Wei et al., 2024). Although classical vaccines such as CV777 were once effective, the emergence of variant strains after 2010 has reduced their protective efficacy, underscoring the need for improved diagnostic tools and a better understanding of viral antigenic features (Wang et al., 2025b,a, 2024).

The nucleocapsid (N) protein is one of the most abundant and conserved proteins of PEDV and is expressed early during infection. These characteristics make it a practical target for serological detection (Zhao et al., 2023). In addition to its structural role in RNA packaging, the N protein has been implicated in viral replication and host-virus interactions, suggesting that its antigenic regions may be subject to functional constraints (Han et al., 2024; Song and Park, 2012; Wang et al., 2016). Compared with the spike (S) protein, which exhibits considerable genetic variability, the relatively conserved nature of the N protein is advantageous for the development of assays with broader strain coverage (Saif, 1993; Song et al., 2023; Zhao et al., 2025). In this study, two minimal linear epitopes (^58^RWRM^61^ and ^15^PLSL^18^) recognized by monoclonal antibodies C9 and G11 were defined using a stepwise truncation strategy. Both epitopes are short, consisting of only four amino acids, indicating that a limited number of residues is sufficient for specific antibody recognition. Similar observations have been reported in other viral systems, where short contiguous motifs represent the core of linear epitopes (Tooze and Stanley, 1986; Lénon et al., 2021; Wu et al., 2017). From a practical standpoint, the identification of such minimal epitopes may facilitate the design of peptide-based reagents and help reduce nonspecific binding in diagnostic assays (Fang et al., 2024; Oka et al., 1994).

As smaller diagnostic antigenic epitope units, tetrapeptides—composed of four amino acids—offer unique advantages in the differential diagnosis of various viruses, including high specificity, low cost, and ease of synthesis. Because they consist of only four amino acids, a mutation in a single amino acid can completely alter their antigenicity, thereby enabling the differentiation of viral variants with over 95% homology. Tetrapeptides designed to target virus-specific conserved regions can avoid cross-reactivity with other pathogens (e.g., PEDV vs. TGEV/PDCoV), thereby achieving high specificity. They can also detect mutations at key sites, such as Leu18 in the PEDV N protein, to distinguish vaccine strains from wild-type strains, as well as highly pathogenic variants from low-pathogenicity variants. The tetrapeptide-based approach shows broad application potential in the differential diagnosis of closely related viruses (such as coronaviruses [e.g., PEDV, SARS-CoV-2] and porcine enteroviruses [e.g., TGEV, PDCoV]), as well as in distinguishing between infection and vaccine-induced immunity (Meloen et al., 1989; Kim et al., 2024).

Sequence alignment showed that both epitopes are highly conserved among representative PEDV strains from different genotypes. This level of conservation suggests that these regions are relatively stable during viral evolution. From an application perspective, conserved epitopes are more suitable for diagnostic assays intended for use across diverse field strains (Tung-Hsuan et al., 2020). Structural modeling further indicated that both epitopes are located on the surface of the N protein. Surface exposure is a typical feature of B-cell epitopes, as it allows direct access for antibody binding (Salvador Eugenio, 2022; Virginie et al., 2010). The agreement between sequence conservation and structural accessibility supports the biological relevance of the identified regions.For the PLSF variant, according to Figure 5F, it is known that mAb G11 cannot maintain good diagnostic sensitivity when facing these PLSF-type strains. However, the L at position 18 of the PEDV N protein is the core essential unit of the NEP-D4 epitope. Existing literature has confirmed that L18 is a necessary amino acid for most monoclonal antibodies to recognize, and its absence completely results in a loss of binding ability. This epitope is conserved at 98.3–100% in different PEDV strains. Therefore, for the vast majority of PEDV strains, mAb G11 continues to demonstrate good effect (Wang et al., 2016; Zhao et al., 2023; Su et al., 2021).

The truncation strategy based on overlapping fragments proved effective in progressively narrowing the epitope regions (Wan-Xiang et al., 2017). Although the recombinant proteins were expressed in Escherichia coli and therefore lack post-translational modifications, this does not affect the identification of linear epitopes (Simson et al., 2023; Airi et al., 2020). Furthermore, predictions using NetNGlyc 1.0 (https://services.healthtech.dtu.dk/services/NetNGlyc-1.0/) indicate that there are no N-linked glycosylation sites near residues 58–61, thereby ruling out any interference from glycan masking effects on the epitope detection experiments in this study; the experimental results reflect solely the antigenic characteristics of the amino acid sequence itself (Zhu et al., 2025; Li et al., 2025b). The results here are consistent with previous studies using similar approaches, supporting the applicability of this method for epitope mapping of viral proteins. At the same time, some limitations of this study should be noted. The epitopes were defined using recombinant fragments, and their performance in the context of native viral infection remains to be evaluated. In addition, potential cross-reactivity with other porcine coronaviruses was not assessed. Further validation using clinical samples would help to clarify their diagnostic utility.

In summary, two conserved and surface-exposed minimal epitopes on the PEDV N protein were identified and characterized. These findings contribute to a more detailed understanding of the antigenic features of the N protein and may be useful for the development of improved serological assays.

## Data Availability

The original contributions presented in the study are included in the article/supplementary material, further inquiries can be directed to the corresponding author/s.
